# 2-(4-Chloro­phen­yl)-5-(cyclo­hex-1-en-1-yl)-3-(4-methyl­phenyl­sulfon­yl)-1-phenyl­imidazolidin-4-one

**DOI:** 10.1107/S1600536811028133

**Published:** 2011-07-23

**Authors:** S. Ranjith, K. SakthiMurugesan, A. SubbiahPandi, K. Namitharan, K. Pitchumani

**Affiliations:** a Department of Physics, Presidency College (Autonomous), Chennai 600 005, India; b School of Chemistry, Madurai Kamaraj University, Madurai 625 021, India

## Abstract

In the title compound, C_28_H_27_ClN_2_O_3_S, the central imidazolidine ring adopts an envelope conformation with the C atom bearing the chloro­phenyl ring at the flap. The geometry around the S atom is distorted tetra­hedral. Three methyl­ene groups of the cyclo­hexene ring are disordered over two sets of sites [site occupancies = 0.562 (10) and 0.438 (10)]. The crystal packing is stabilized by C—H⋯π inter­actions.

## Related literature

For the biological activity of sulfonamides, see: Zareef *et al.* (2007 ▶); Chohan & Shad (2008 ▶); Pomarnacka & Kozlarska-Kedra (2003 ▶); Nieto *et al.* (2005 ▶); Wang *et al.* (1995 ▶). For a related structure, see: Ranjith *et al.* (2011 ▶). For puckering parameters, see: Cremer & Pople (1975 ▶). For ring asymmetry parameters, see: Nardelli *et al.* (1983 ▶).
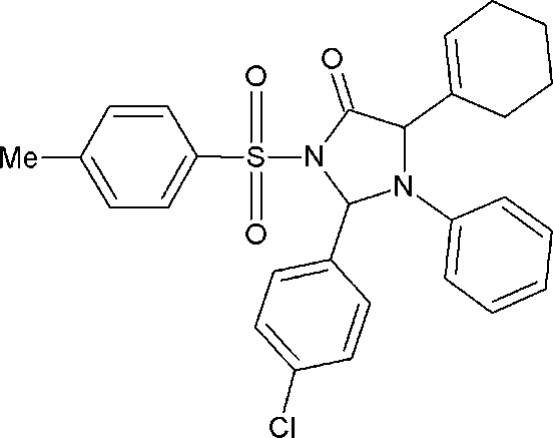


## Experimental

### 

#### Crystal data

C_28_H_27_ClN_2_O_3_S
*M* *_r_* = 507.03
Monoclinic, 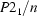

*a* = 10.9974 (3) Å
*b* = 13.4095 (4) Å
*c* = 17.4434 (5) Å
β = 105.103 (2)°
*V* = 2483.52 (12) Å^3^
*Z* = 4
Mo *K*α radiation
μ = 0.27 mm^−1^
*T* = 293 K
0.25 × 0.22 × 0.19 mm

#### Data collection

Bruker APEXII CCD area detector diffractometer
Absorption correction: multi-scan (*SADABS*; Sheldrick, 1996 ▶) *T*_min_ = 0.934, *T*_max_ = 0.950
32488 measured reflections
7366 independent reflections
4844 reflections with *I* > 2σ(*I*)
*R*_int_ = 0.034

#### Refinement

*R*[*F* ^2^ > 2σ(*F* ^2^)] = 0.047
*wR*(*F* ^2^) = 0.139
*S* = 1.01
7366 reflections
346 parameters
4 restraints
H-atom parameters constrained
Δρ_max_ = 0.30 e Å^−3^
Δρ_min_ = −0.33 e Å^−3^

### 

Data collection: *APEX2* (Bruker, 2004 ▶); cell refinement: *SAINT* (Bruker, 2004 ▶); data reduction: *SAINT*; program(s) used to solve structure: *SHELXS97* (Sheldrick, 2008 ▶); program(s) used to refine structure: *SHELXL97* (Sheldrick, 2008 ▶); molecular graphics: *ORTEP-3* (Farrugia, 1997 ▶); software used to prepare material for publication: *SHELXL97* and *PLATON* (Spek, 2009 ▶).

## Supplementary Material

Crystal structure: contains datablock(s) global, I. DOI: 10.1107/S1600536811028133/bt5573sup1.cif

Structure factors: contains datablock(s) I. DOI: 10.1107/S1600536811028133/bt5573Isup2.hkl

Supplementary material file. DOI: 10.1107/S1600536811028133/bt5573Isup3.cml

Additional supplementary materials:  crystallographic information; 3D view; checkCIF report

## Figures and Tables

**Table 1:** Hydrogen-bond geometry (Å, °) *Cg*2 and *Cg*4 are the centroids of the C2–C7 and C15–C20 rings, respectively.

*D*—H⋯*A*	*D*—H	H⋯*A*	*D*⋯*A*	*D*—H⋯*A*
C1—H1*A*⋯*Cg*4^i^	0.96	2.91	3.490 (3)	120
C11—H11⋯*Cg*2^i^	0.93	2.86	3.612 (2)	139

Symmetry code: (i) 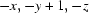.

**Fig. 1.**
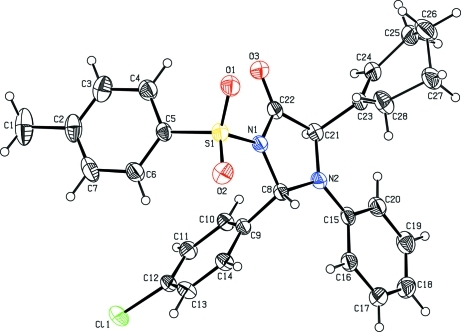
The structure of showing the atom-numbering scheme. The displacement ellipsoids are drawn at the 30% probability level. The disordered atoms are omitted for clarity.

## supplementary crystallographic information

### Comment

Sulfonamides have widely been recognized for their wide variety of pharmacological activities such as antibacterial, antitumor, anti-carbonic anhydrase, diuretic, hypoglycaemic, antithyroid and protease inhibitory activity. Sulfonamides have also been used clinically as antimalarial agents (Zareef *et al.*, 2007), particularly sulfadiazine and sulfadoxine. Due to their significant pharmacology applications and widespread use in medicine, these compounds have also gained attention in bioinorganic and metal-based (Chohan *et al.*, 2008) drug chemistry. Sulfonamide derivatives are well known drugs and are used to control diseases caused by bacterial infections. Benzene sulfonamide derivatives are known to exhibit anticancer and HIV activities (Pomarnacka & Kozlarska-Kedra, 2003) and antibacterial activities (Nieto *et al.*, 2005). Imidazolidine compounds are important intermediates and building blocks in the construction of various biologically active compounds (Wang *et al.*, 1995). Against this background, and in order to obtain detailed information on molecular conformations in the solid state, an X-ray study of the title compound was carried out.

X-Ray analysis confirms the molecular structure and atom connectivity as illustrated in Fig. 1. The geometry around the S atom is distorted tetrahedral, comprising two O atoms of the sulfonyl group, a C atom of a phenyl ring and the imidazolidine N atom. The S–O, S–C, and S–N distances are 1.416 (2), 1.747 (2) and 1.677 (2) Å, respectively, these are comparable as observed in similar structures (Ranjith *et al.*, 2011). The atom Cl1 is deviated by 0.136 (1)Å from the leastsquares plane of the rings C9–C14. The S atom exhibits significant deviation from that of a regular tetrahedron, with the largest deviations for the O–S–O [O1–S1–O2 121.2 (9)°] and O–S–N angles [O1–S1–N1 106.4 (7)°]. The widening of the angles may be due to repulsive interactions between the two short S=O bonds, similar to what is observed in related structures (Ranjith *et al.*, 2011).

The imidazolidine ring adopts envelope conformation, with the puckering parameters q_2_ and φ (Cremer & Pople, 1975) and the smallest displacement asymmetric parameters,Δ, (Nardelli *et al.*, 1983) as follows: q_2_=0.1230 (17) Å, φ=211.7 (8)°, Δ_s_(C8)=1.50 (17) and the cyclohexane ring adopts half-chair conformation, in addition to with the puckering parameters q_2_ and φ (Cremer & Pople, 1975) and the smallest displacement asymmetric parameters,Δ, (Nardelli *et al.*, 1983) as follows: q_2_=0.399 (7) Å, φ=127.2 (8)°, Δ_s_(C24)= 18.4 (7). In the crystal, the molecules form layers that are linked by π···π stacking interactions between the imidazolidine and benzene [C9—C14] rings [centroid–centroid distances = 3.7406 (9) Å].

### Experimental

4-Toluenesulfonyl azide (1.3 mmol), 1-ethynyl cyclohexene (1.2 mmol), 4-chlorophenyl *N*-phenylnitrone (1.0 mmol) and triethylamine (2 mmol) were successively added to Cu^1^—Y zeolite (30 mg) in dichloromethane under N_2_ atmosphere. After stirring at room temperature for the 6 h, the mixture was diluted with dichloromethane. After removing the catalyst by filtration, followed by solvent evaporation, the resulting crude product was finally purified by column chromatography (silica gel). Single crystals suitable for X-ray diffraction were obtained by slow evaporation of a solution of the title compound in acetone at room temperature.

### Refinement

Three methylene groups of the cyclohexane ring are disordered over two positions (C25/C25', C26/C26' and C27/C27') with refined occupancies of 0.562 (10) and 0.438 (10). The corresponding bond distances involving the disordered atoms were restrained to be equal. All H atoms were fixed geometrically and allowed to ride on their parent C atoms, with C—H distances fixed in the range 0.93–0.97 Å with *U*_iso_(H) = 1.5*U*_eq_(C) for methyl H 1.2*U*_eq_(C) for other H atoms.

### Crystal data

**Table:**

C_28_H_27_ClN_2_O_3_S	*F*(000) = 1064
*M**_r_* = 507.03	*D*_x_ = 1.356 Mg m^−^^3^
Monoclinic, *P*2_1_/*n*	Mo *K*α radiation, λ = 0.71073 Å
Hall symbol: -P 2yn	Cell parameters from 7366 reflections
*a* = 10.9974 (3) Å	θ = 2.0–30.4°
*b* = 13.4095 (4) Å	µ = 0.27 mm^−^^1^
*c* = 17.4434 (5) Å	*T* = 293 K
β = 105.103 (2)°	Block, white crystalline
*V* = 2483.52 (12) Å^3^	0.25 × 0.22 × 0.19 mm
*Z* = 4	

### Data collection

**Table:**

Bruker APEXII CCD area detector diffractometer	7366 independent reflections
Radiation source: fine-focus sealed tube	4844 reflections with *I* > 2σ(*I*)
graphite	*R*_int_ = 0.034
ω and φ scans	θ_max_ = 30.4°, θ_min_ = 2.0°
Absorption correction: multi-scan (*SADABS*; Sheldrick, 1996)	*h* = −15→15
*T*_min_ = 0.934, *T*_max_ = 0.950	*k* = −18→17
32488 measured reflections	*l* = −24→24

### Refinement

**Table:**

Refinement on *F*^2^	Secondary atom site location: difference Fourier map
Least-squares matrix: full	Hydrogen site location: inferred from neighbouring sites
*R*[*F*^2^ > 2σ(*F*^2^)] = 0.047	H-atom parameters constrained
*wR*(*F*^2^) = 0.139	*w* = 1/[σ^2^(*F*_o_^2^) + (0.0638*P*)^2^ + 0.6285*P*] where *P* = (*F*_o_^2^ + 2*F*_c_^2^)/3
*S* = 1.01	(Δ/σ)_max_ < 0.001
7366 reflections	Δρ_max_ = 0.30 e Å^−^^3^
346 parameters	Δρ_min_ = −0.33 e Å^−^^3^
4 restraints	Extinction correction: *SHELXL97* (Sheldrick, 2008), Fc^*^=kFc[1+0.001xFc^2^λ^3^/sin(2θ)]^-1/4^
Primary atom site location: structure-invariant direct methods	Extinction coefficient: 0.0016 (5)

### Special details

**Table:**

Geometry. All e.s.d.'s (except the e.s.d. in the dihedral angle between two l.s. planes) are estimated using the full covariance matrix. The cell e.s.d.'s are taken into account individually in the estimation of e.s.d.'s in distances, angles and torsion angles; correlations between e.s.d.'s in cell parameters are only used when they are defined by crystal symmetry. An approximate (isotropic) treatment of cell e.s.d.'s is used for estimating e.s.d.'s involving l.s. planes.
Refinement. Refinement of *F*^2^ against ALL reflections. The weighted *R*-factor *wR* and goodness of fit *S* are based on *F*^2^, conventional *R*-factors *R* are based on *F*, with *F* set to zero for negative *F*^2^. The threshold expression of *F*^2^ > σ(*F*^2^) is used only for calculating *R*-factors(gt) *etc*. and is not relevant to the choice of reflections for refinement. *R*-factors based on *F*^2^ are statistically about twice as large as those based on *F*, and *R*- factors based on ALL data will be even larger.

### Fractional atomic coordinates and isotropic or equivalent isotropic displacement parameters (Å^2^)

**Table:**

	*x*	*y*	*z*	*U*_iso_*/*U*_eq_	Occ. (<1)
C1	0.3220 (3)	0.3780 (3)	−0.10334 (16)	0.0984 (11)	
H1A	0.2431	0.3432	−0.1139	0.148*	
H1B	0.3896	0.3306	−0.0959	0.148*	
H1C	0.3229	0.4206	−0.1474	0.148*	
C2	0.3385 (2)	0.4401 (2)	−0.02909 (13)	0.0630 (6)	
C3	0.3809 (2)	0.39928 (18)	0.04443 (15)	0.0657 (6)	
H3	0.4018	0.3319	0.0488	0.079*	
C4	0.3938 (2)	0.45477 (15)	0.11277 (12)	0.0534 (5)	
H4	0.4217	0.4250	0.1624	0.064*	
C5	0.36483 (15)	0.55480 (14)	0.10642 (10)	0.0399 (4)	
C6	0.32259 (19)	0.59833 (18)	0.03261 (11)	0.0551 (5)	
H6	0.3030	0.6659	0.0279	0.066*	
C7	0.3098 (2)	0.5397 (2)	−0.03421 (12)	0.0683 (7)	
H7	0.2809	0.5687	−0.0841	0.082*	
C8	0.15703 (15)	0.68904 (13)	0.20985 (9)	0.0357 (3)	
H8	0.1924	0.7545	0.2284	0.043*	
C9	0.08827 (14)	0.69380 (12)	0.12235 (9)	0.0335 (3)	
C10	0.01018 (16)	0.61671 (13)	0.08682 (10)	0.0393 (4)	
H10	−0.0062	0.5645	0.1178	0.047*	
C11	−0.04370 (16)	0.61627 (14)	0.00593 (10)	0.0428 (4)	
H11	−0.0969	0.5646	−0.0176	0.051*	
C12	−0.01773 (16)	0.69310 (14)	−0.03927 (9)	0.0420 (4)	
C13	0.05538 (18)	0.77282 (14)	−0.00504 (10)	0.0467 (4)	
H13	0.0696	0.8257	−0.0361	0.056*	
C14	0.10737 (17)	0.77306 (13)	0.07623 (10)	0.0412 (4)	
H14	0.1557	0.8271	0.1001	0.049*	
C15	−0.02845 (16)	0.69953 (14)	0.26500 (9)	0.0392 (4)	
C16	−0.05654 (17)	0.79523 (14)	0.23440 (10)	0.0450 (4)	
H16	−0.0010	0.8280	0.2109	0.054*	
C17	−0.1665 (2)	0.84149 (17)	0.23890 (12)	0.0558 (5)	
H17	−0.1839	0.9057	0.2188	0.067*	
C18	−0.2509 (2)	0.79421 (19)	0.27261 (12)	0.0606 (6)	
H18	−0.3255	0.8255	0.2744	0.073*	
C19	−0.22360 (19)	0.70099 (19)	0.30332 (12)	0.0585 (5)	
H19	−0.2799	0.6691	0.3267	0.070*	
C20	−0.11374 (18)	0.65312 (16)	0.30031 (11)	0.0498 (4)	
H20	−0.0965	0.5897	0.3219	0.060*	
C21	0.11459 (17)	0.55093 (13)	0.29037 (9)	0.0407 (4)	
H21	0.0469	0.5047	0.2652	0.049*	
C22	0.23143 (17)	0.52896 (14)	0.26277 (9)	0.0416 (4)	
C23	0.14710 (17)	0.53918 (13)	0.37990 (9)	0.0401 (4)	
N1	0.25822 (13)	0.61314 (11)	0.22405 (8)	0.0383 (3)	
N2	0.08217 (14)	0.65180 (11)	0.26115 (8)	0.0416 (3)	
O1	0.48872 (12)	0.58412 (12)	0.25198 (8)	0.0570 (4)	
O2	0.38986 (13)	0.72903 (10)	0.17229 (9)	0.0552 (3)	
O3	0.29106 (14)	0.45255 (10)	0.27358 (8)	0.0552 (3)	
S1	0.38901 (4)	0.62668 (4)	0.19262 (2)	0.04154 (13)	
Cl1	−0.07606 (6)	0.68766 (5)	−0.14158 (3)	0.06699 (18)	
C24	0.1128 (2)	0.45825 (15)	0.41165 (11)	0.0495 (4)	
H24	0.0584	0.4123	0.3803	0.059*	
C28	0.2297 (2)	0.61722 (17)	0.42755 (11)	0.0626 (6)	
H28A	0.1981	0.6823	0.4074	0.075*	
H28B	0.3137	0.6102	0.4202	0.075*	
C25	0.1632 (15)	0.4414 (10)	0.4994 (2)	0.062 (3)	0.562 (10)
H25A	0.1889	0.3723	0.5081	0.074*	0.562 (10)
H25B	0.0956	0.4525	0.5247	0.074*	0.562 (10)
C26	0.2734 (7)	0.5069 (4)	0.5394 (3)	0.070 (2)	0.562 (10)
H26A	0.2921	0.4992	0.5966	0.083*	0.562 (10)
H26B	0.3475	0.4881	0.5225	0.083*	0.562 (10)
C27	0.2384 (8)	0.6130 (3)	0.5167 (2)	0.0693 (18)	0.562 (10)
H27A	0.3023	0.6585	0.5460	0.083*	0.562 (10)
H27B	0.1582	0.6300	0.5268	0.083*	0.562 (10)
C25'	0.1412 (18)	0.4256 (11)	0.4969 (3)	0.052 (2)	0.438 (10)
H25C	0.1931	0.3661	0.5050	0.062*	0.438 (10)
H25D	0.0637	0.4109	0.5113	0.062*	0.438 (10)
C26'	0.2104 (10)	0.5104 (8)	0.5469 (5)	0.075 (3)	0.438 (10)
H26C	0.1488	0.5540	0.5605	0.090*	0.438 (10)
H26D	0.2621	0.4829	0.5961	0.090*	0.438 (10)
C27'	0.2928 (6)	0.5719 (8)	0.5090 (3)	0.092 (4)	0.438 (10)
H27C	0.3623	0.5306	0.5035	0.111*	0.438 (10)
H27D	0.3281	0.6259	0.5450	0.111*	0.438 (10)

### Atomic displacement parameters (Å^2^)

**Table:**

	*U*^11^	*U*^22^	*U*^33^	*U*^12^	*U*^13^	*U*^23^
C1	0.0785 (18)	0.152 (3)	0.0741 (17)	−0.0432 (18)	0.0375 (14)	−0.0598 (18)
C2	0.0472 (11)	0.0952 (18)	0.0520 (12)	−0.0222 (12)	0.0228 (9)	−0.0241 (12)
C3	0.0725 (15)	0.0610 (13)	0.0723 (15)	−0.0144 (11)	0.0345 (12)	−0.0184 (11)
C4	0.0604 (12)	0.0556 (12)	0.0468 (10)	−0.0013 (10)	0.0186 (9)	0.0022 (9)
C5	0.0349 (8)	0.0528 (10)	0.0340 (8)	−0.0017 (7)	0.0124 (6)	0.0003 (7)
C6	0.0549 (11)	0.0706 (14)	0.0416 (10)	0.0078 (10)	0.0159 (8)	0.0099 (9)
C7	0.0568 (12)	0.117 (2)	0.0324 (9)	−0.0009 (13)	0.0133 (9)	0.0025 (11)
C8	0.0349 (8)	0.0439 (9)	0.0279 (7)	−0.0005 (7)	0.0077 (6)	−0.0007 (6)
C9	0.0319 (7)	0.0399 (9)	0.0290 (7)	0.0002 (6)	0.0082 (6)	0.0011 (6)
C10	0.0420 (9)	0.0409 (9)	0.0345 (8)	−0.0053 (7)	0.0091 (7)	0.0022 (7)
C11	0.0399 (9)	0.0457 (10)	0.0391 (9)	−0.0019 (7)	0.0037 (7)	−0.0053 (7)
C12	0.0423 (9)	0.0531 (10)	0.0276 (7)	0.0079 (8)	0.0039 (6)	0.0011 (7)
C13	0.0529 (10)	0.0473 (10)	0.0382 (9)	−0.0002 (8)	0.0085 (8)	0.0112 (7)
C14	0.0435 (9)	0.0398 (9)	0.0382 (8)	−0.0059 (7)	0.0068 (7)	0.0023 (7)
C15	0.0369 (8)	0.0552 (10)	0.0246 (7)	0.0012 (7)	0.0062 (6)	−0.0038 (7)
C16	0.0458 (10)	0.0537 (11)	0.0356 (8)	0.0012 (8)	0.0108 (7)	−0.0030 (7)
C17	0.0557 (12)	0.0637 (13)	0.0466 (10)	0.0147 (10)	0.0110 (9)	−0.0020 (9)
C18	0.0450 (11)	0.0851 (16)	0.0516 (11)	0.0149 (11)	0.0121 (9)	−0.0055 (11)
C19	0.0423 (10)	0.0897 (17)	0.0468 (10)	−0.0004 (10)	0.0174 (8)	0.0024 (10)
C20	0.0451 (10)	0.0666 (12)	0.0390 (9)	0.0030 (9)	0.0134 (8)	0.0060 (8)
C21	0.0455 (9)	0.0469 (10)	0.0306 (8)	−0.0008 (8)	0.0114 (7)	0.0013 (7)
C22	0.0468 (10)	0.0495 (10)	0.0284 (7)	0.0019 (8)	0.0095 (7)	0.0012 (7)
C23	0.0478 (9)	0.0443 (9)	0.0297 (7)	0.0018 (8)	0.0128 (7)	0.0017 (7)
N1	0.0366 (7)	0.0478 (8)	0.0314 (6)	0.0043 (6)	0.0103 (5)	0.0039 (6)
N2	0.0430 (8)	0.0506 (8)	0.0350 (7)	0.0067 (7)	0.0169 (6)	0.0086 (6)
O1	0.0382 (7)	0.0846 (10)	0.0426 (7)	0.0081 (7)	0.0006 (5)	−0.0051 (7)
O2	0.0499 (8)	0.0530 (8)	0.0662 (9)	−0.0085 (6)	0.0217 (7)	−0.0033 (7)
O3	0.0656 (9)	0.0516 (8)	0.0523 (8)	0.0137 (7)	0.0225 (7)	0.0076 (6)
S1	0.0334 (2)	0.0542 (3)	0.0366 (2)	−0.00127 (18)	0.00827 (16)	−0.00333 (18)
Cl1	0.0809 (4)	0.0804 (4)	0.0305 (2)	0.0147 (3)	−0.0021 (2)	0.0009 (2)
C24	0.0613 (11)	0.0503 (11)	0.0409 (9)	0.0044 (9)	0.0207 (8)	0.0039 (8)
C28	0.0794 (15)	0.0635 (14)	0.0400 (10)	−0.0123 (11)	0.0065 (10)	−0.0029 (9)
C25	0.093 (8)	0.054 (4)	0.045 (3)	−0.002 (3)	0.030 (3)	0.012 (3)
C26	0.094 (5)	0.076 (3)	0.033 (2)	0.006 (4)	0.006 (3)	0.004 (2)
C27	0.106 (5)	0.064 (3)	0.037 (2)	0.004 (3)	0.016 (2)	−0.0115 (18)
C25'	0.064 (5)	0.052 (5)	0.044 (4)	0.006 (3)	0.024 (3)	0.021 (3)
C26'	0.099 (7)	0.090 (5)	0.042 (3)	−0.016 (5)	0.029 (4)	0.010 (3)
C27'	0.101 (6)	0.137 (10)	0.036 (3)	−0.068 (6)	0.013 (3)	−0.004 (4)

### Geometric parameters (Å, °)

**Table:**

C1—C2	1.511 (3)	C18—H18	0.9300
C1—H1A	0.9600	C19—C20	1.381 (3)
C1—H1B	0.9600	C19—H19	0.9300
C1—H1C	0.9600	C20—H20	0.9300
C2—C3	1.360 (3)	C21—N2	1.457 (2)
C2—C7	1.370 (4)	C21—C22	1.514 (2)
C3—C4	1.381 (3)	C21—C23	1.517 (2)
C3—H3	0.9300	C21—H21	0.9800
C4—C5	1.376 (3)	C22—O3	1.204 (2)
C4—H4	0.9300	C22—N1	1.386 (2)
C5—C6	1.379 (2)	C23—C24	1.317 (3)
C5—S1	1.7471 (17)	C23—C28	1.489 (3)
C6—C7	1.382 (3)	N1—S1	1.6774 (14)
C6—H6	0.9300	O1—S1	1.4173 (13)
C7—H7	0.9300	O2—S1	1.4181 (15)
C8—N2	1.453 (2)	C24—C25	1.502 (3)
C8—N1	1.480 (2)	C24—C25'	1.502 (3)
C8—C9	1.518 (2)	C24—H24	0.9300
C8—H8	0.9800	C28—C27	1.534 (3)
C9—C14	1.382 (2)	C28—C27'	1.535 (4)
C9—C10	1.383 (2)	C28—H28A	0.9700
C10—C11	1.381 (2)	C28—H28B	0.9700
C10—H10	0.9300	C25—C26	1.511 (6)
C11—C12	1.372 (3)	C25—H25A	0.9700
C11—H11	0.9300	C25—H25B	0.9700
C12—C13	1.377 (3)	C26—C27	1.501 (5)
C12—Cl1	1.7335 (16)	C26—H26A	0.9700
C13—C14	1.384 (2)	C26—H26B	0.9700
C13—H13	0.9300	C27—H27A	0.9700
C14—H14	0.9300	C27—H27B	0.9700
C15—N2	1.392 (2)	C25'—C26'	1.511 (6)
C15—C16	1.393 (3)	C25'—H25C	0.9700
C15—C20	1.395 (3)	C25'—H25D	0.9700
C16—C17	1.380 (3)	C26'—C27'	1.501 (5)
C16—H16	0.9300	C26'—H26C	0.9700
C17—C18	1.375 (3)	C26'—H26D	0.9700
C17—H17	0.9300	C27'—H27C	0.9700
C18—C19	1.362 (3)	C27'—H27D	0.9700
			
C2—C1—H1A	109.5	C22—C21—H21	110.1
C2—C1—H1B	109.5	C23—C21—H21	110.1
H1A—C1—H1B	109.5	O3—C22—N1	126.36 (16)
C2—C1—H1C	109.5	O3—C22—C21	126.04 (16)
H1A—C1—H1C	109.5	N1—C22—C21	107.59 (15)
H1B—C1—H1C	109.5	C24—C23—C28	122.96 (16)
C3—C2—C7	118.08 (19)	C24—C23—C21	120.13 (17)
C3—C2—C1	121.4 (3)	C28—C23—C21	116.58 (15)
C7—C2—C1	120.5 (2)	C22—N1—C8	113.30 (13)
C2—C3—C4	122.0 (2)	C22—N1—S1	123.83 (12)
C2—C3—H3	119.0	C8—N1—S1	122.82 (11)
C4—C3—H3	119.0	C15—N2—C8	120.95 (14)
C5—C4—C3	119.1 (2)	C15—N2—C21	123.20 (14)
C5—C4—H4	120.5	C8—N2—C21	114.10 (13)
C3—C4—H4	120.5	O1—S1—O2	121.21 (9)
C4—C5—C6	120.15 (18)	O1—S1—N1	106.48 (8)
C4—C5—S1	119.22 (14)	O2—S1—N1	104.40 (8)
C6—C5—S1	120.56 (15)	O1—S1—C5	108.78 (9)
C5—C6—C7	118.8 (2)	O2—S1—C5	109.16 (9)
C5—C6—H6	120.6	N1—S1—C5	105.69 (7)
C7—C6—H6	120.6	C23—C24—C25	118.8 (4)
C2—C7—C6	121.9 (2)	C23—C24—C25'	130.3 (4)
C2—C7—H7	119.1	C23—C24—H24	120.6
C6—C7—H7	119.1	C25—C24—H24	120.6
N2—C8—N1	100.51 (12)	C25'—C24—H24	108.9
N2—C8—C9	114.83 (13)	C23—C28—C27	114.1 (3)
N1—C8—C9	110.67 (13)	C23—C28—C27'	107.5 (4)
N2—C8—H8	110.2	C23—C28—H28A	108.7
N1—C8—H8	110.2	C27—C28—H28A	108.7
C9—C8—H8	110.2	C27'—C28—H28A	136.3
C14—C9—C10	119.00 (14)	C23—C28—H28B	108.7
C14—C9—C8	120.42 (14)	C27—C28—H28B	108.7
C10—C9—C8	120.51 (14)	C27'—C28—H28B	82.4
C11—C10—C9	120.81 (16)	H28A—C28—H28B	107.6
C11—C10—H10	119.6	C24—C25—C26	115.0 (5)
C9—C10—H10	119.6	C24—C25—H25A	108.5
C12—C11—C10	119.08 (16)	C26—C25—H25A	108.5
C12—C11—H11	120.5	C24—C25—H25B	108.5
C10—C11—H11	120.5	C26—C25—H25B	108.5
C11—C12—C13	121.28 (15)	H25A—C25—H25B	107.5
C11—C12—Cl1	118.91 (14)	C27—C26—C25	108.0 (8)
C13—C12—Cl1	119.79 (14)	C27—C26—H26A	110.1
C12—C13—C14	119.01 (16)	C25—C26—H26A	110.1
C12—C13—H13	120.5	C27—C26—H26B	110.1
C14—C13—H13	120.5	C25—C26—H26B	110.1
C9—C14—C13	120.66 (16)	H26A—C26—H26B	108.4
C9—C14—H14	119.7	C26—C27—C28	104.3 (3)
C13—C14—H14	119.7	C26—C27—H27A	110.9
N2—C15—C16	120.96 (16)	C28—C27—H27A	110.9
N2—C15—C20	120.84 (17)	C26—C27—H27B	110.9
C16—C15—C20	118.19 (17)	C28—C27—H27B	110.9
C17—C16—C15	120.16 (18)	H27A—C27—H27B	108.9
C17—C16—H16	119.9	C24—C25'—C26'	107.2 (6)
C15—C16—H16	119.9	C24—C25'—H25C	110.3
C18—C17—C16	121.0 (2)	C26'—C25'—H25C	110.3
C18—C17—H17	119.5	C24—C25'—H25D	110.3
C16—C17—H17	119.5	C26'—C25'—H25D	110.3
C19—C18—C17	119.2 (2)	H25C—C25'—H25D	108.5
C19—C18—H18	120.4	C27'—C26'—C25'	115.5 (9)
C17—C18—H18	120.4	C27'—C26'—H26C	108.4
C18—C19—C20	121.1 (2)	C25'—C26'—H26C	108.4
C18—C19—H19	119.5	C27'—C26'—H26D	108.4
C20—C19—H19	119.5	C25'—C26'—H26D	108.4
C19—C20—C15	120.3 (2)	H26C—C26'—H26D	107.5
C19—C20—H20	119.8	C26'—C27'—C28	116.5 (6)
C15—C20—H20	119.8	C26'—C27'—H27C	108.2
N2—C21—C22	102.71 (13)	C28—C27'—H27C	108.2
N2—C21—C23	115.29 (14)	C26'—C27'—H27D	108.2
C22—C21—C23	108.38 (14)	C28—C27'—H27D	108.2
N2—C21—H21	110.1	H27C—C27'—H27D	107.3
			
C7—C2—C3—C4	0.8 (3)	N2—C8—N1—S1	−169.07 (11)
C1—C2—C3—C4	−178.6 (2)	C9—C8—N1—S1	69.17 (16)
C2—C3—C4—C5	−1.0 (3)	C16—C15—N2—C8	−14.4 (2)
C3—C4—C5—C6	0.5 (3)	C20—C15—N2—C8	166.21 (16)
C3—C4—C5—S1	−176.61 (16)	C16—C15—N2—C21	−178.39 (15)
C4—C5—C6—C7	0.2 (3)	C20—C15—N2—C21	2.2 (2)
S1—C5—C6—C7	177.24 (16)	N1—C8—N2—C15	−177.89 (14)
C3—C2—C7—C6	−0.1 (3)	C9—C8—N2—C15	−59.1 (2)
C1—C2—C7—C6	179.3 (2)	N1—C8—N2—C21	−12.53 (17)
C5—C6—C7—C2	−0.4 (3)	C9—C8—N2—C21	106.25 (16)
N2—C8—C9—C14	140.82 (16)	C22—C21—N2—C15	172.55 (14)
N1—C8—C9—C14	−106.26 (18)	C23—C21—N2—C15	−69.8 (2)
N2—C8—C9—C10	−42.2 (2)	C22—C21—N2—C8	7.56 (18)
N1—C8—C9—C10	70.73 (19)	C23—C21—N2—C8	125.21 (15)
C14—C9—C10—C11	2.9 (3)	C22—N1—S1—O1	−38.92 (15)
C8—C9—C10—C11	−174.14 (16)	C8—N1—S1—O1	143.77 (13)
C9—C10—C11—C12	0.7 (3)	C22—N1—S1—O2	−168.25 (14)
C10—C11—C12—C13	−3.4 (3)	C8—N1—S1—O2	14.44 (14)
C10—C11—C12—Cl1	175.27 (14)	C22—N1—S1—C5	76.66 (15)
C11—C12—C13—C14	2.5 (3)	C8—N1—S1—C5	−100.65 (13)
Cl1—C12—C13—C14	−176.16 (15)	C4—C5—S1—O1	28.34 (18)
C10—C9—C14—C13	−3.8 (3)	C6—C5—S1—O1	−148.76 (15)
C8—C9—C14—C13	173.23 (16)	C4—C5—S1—O2	162.56 (15)
C12—C13—C14—C9	1.1 (3)	C6—C5—S1—O2	−14.54 (17)
N2—C15—C16—C17	−179.85 (16)	C4—C5—S1—N1	−85.66 (16)
C20—C15—C16—C17	−0.4 (2)	C6—C5—S1—N1	97.24 (16)
C15—C16—C17—C18	−0.7 (3)	C28—C23—C24—C25	−2.5 (9)
C16—C17—C18—C19	1.3 (3)	C21—C23—C24—C25	170.6 (9)
C17—C18—C19—C20	−0.7 (3)	C28—C23—C24—C25'	1.8 (12)
C18—C19—C20—C15	−0.4 (3)	C21—C23—C24—C25'	174.9 (12)
N2—C15—C20—C19	−179.59 (17)	C24—C23—C28—C27	−17.9 (4)
C16—C15—C20—C19	1.0 (3)	C21—C23—C28—C27	168.7 (3)
N2—C21—C22—O3	−179.64 (17)	C24—C23—C28—C27'	15.9 (5)
C23—C21—C22—O3	57.9 (2)	C21—C23—C28—C27'	−157.5 (4)
N2—C21—C22—N1	1.29 (17)	C23—C24—C25—C26	−14.2 (17)
C23—C21—C22—N1	−121.16 (15)	C25'—C24—C25—C26	−178 (9)
N2—C21—C23—C24	143.30 (18)	C24—C25—C26—C27	50.9 (15)
C22—C21—C23—C24	−102.3 (2)	C25—C26—C27—C28	−67.4 (9)
N2—C21—C23—C28	−43.1 (2)	C23—C28—C27—C26	52.2 (7)
C22—C21—C23—C28	71.3 (2)	C27'—C28—C27—C26	−32.6 (5)
O3—C22—N1—C8	171.37 (16)	C23—C24—C25'—C26'	6(2)
C21—C22—N1—C8	−9.56 (18)	C25—C24—C25'—C26'	24 (7)
O3—C22—N1—S1	−6.2 (3)	C24—C25'—C26'—C27'	−32.2 (19)
C21—C22—N1—S1	172.90 (11)	C25'—C26'—C27'—C28	54.7 (17)
N2—C8—N1—C22	13.37 (17)	C23—C28—C27'—C26'	−42.8 (12)
C9—C8—N1—C22	−108.40 (15)	C27—C28—C27'—C26'	64.7 (9)

### Hydrogen-bond geometry (Å, °)

**Table:**

Cg2 and Cg4 are the centroids of the C2–C7 and C15–C20 rings, respectively.

**Table:**

*D*—H···*A*	*D*—H	H···*A*	*D*···*A*	*D*—H···*A*
C1—H1A···Cg4^i^	0.96	2.91	3.490 (3)	120
C11—H11···Cg2^i^	0.93	2.86	3.612 (2)	139

Symmetry codes: (i) −*x*, −*y*+1, −*z*.

## References

[bb1] Bruker (2004). *APEX2* and *SAINT* Bruker AXS Inc., Madison, Wisconsin, USA.

[bb2] Chohan, Z. H. & Shad, H. A. (2008). *J. Enz. Inhib. Med. Chem.* **23**, 369–379. 10.1080/14756360701585692 18569342

[bb3] Cremer, D. & Pople, J. A. (1975). *J. Am. Chem. Soc.* **97**, 1354–1358.

[bb4] Farrugia, L. J. (1997). *J. Appl. Cryst.* **30**, 565.

[bb5] Nardelli, M. (1983). *Acta Cryst.* C**39**, 1141–1142.

[bb6] Nieto, M. J., Alovero, F. L., Manzo, R. H. & Mazzieri, M. R. (2005). *Eur. J. Med. Chem.* **40**, 361–369. 10.1016/j.ejmech.2004.11.008 15804535

[bb7] Pomarnacka, E. & Kozlarska-Kedra, I. (2003). *Farmaco*, **58**, 423–429. 10.1016/S0014-827X(03)00071-5 12767381

[bb8] Ranjith, S., SubbiahPandi, A., Namitharan, K. & Pitchumani, K. (2011). *Acta Cryst.* E**67**, o843. 10.1107/S1600536811008427 PMC3099910 21754125

[bb9] Sheldrick, G. M. (1996). *SADABS* University of Göttingen, Germany.

[bb10] Sheldrick, G. M. (2008). *Acta Cryst.* A**64**, 112–122. 10.1107/S0108767307043930 18156677

[bb11] Spek, A. L. (2009). *Acta Cryst.* D**65**, 148–155. 10.1107/S090744490804362X PMC2631630 19171970

[bb12] Wang, W., Liang, T. C., Zheng, M. & Gao, X. (1995). *Tetrahedron Lett.* **36**, 1181–1184.

[bb13] Zareef, M., Iqbal, R., De Dominguez, N. G., Rodrigues, J., Zaidi, J. H., Arfan, M. & Supuran, C. T. (2007). *J. Enz. Inhib. Med. Chem.* **22**, 301–308. 10.1080/14756360601114569 17674812

